# Scalable Sparse Testing Genomic Selection Strategy for Early Yield Testing Stage

**DOI:** 10.3389/fpls.2021.658978

**Published:** 2021-06-22

**Authors:** Sikiru Adeniyi Atanda, Michael Olsen, Jose Crossa, Juan Burgueño, Renaud Rincent, Daniel Dzidzienyo, Yoseph Beyene, Manje Gowda, Kate Dreher, Prasanna M. Boddupalli, Pangirayi Tongoona, Eric Yirenkyi Danquah, Gbadebo Olaoye, Kelly R. Robbins

**Affiliations:** ^1^West Africa Center for Crop Improvement (WACCI), University of Ghana, Accra, Ghana; ^2^International Maize and Wheat Improvement Center (CIMMYT), Texcoco, Mexico; ^3^Section of Plant Breeding and Genetics, School of Integrative Plant Sciences, Cornell University, Ithaca, NY, United States; ^4^International Maize and Wheat Improvement Center (CIMMYT), Nairobi, Kenya; ^5^French National Institute for Agriculture, Food, and Environment (INRAE), Paris, France; ^6^Agronomy Department, University of Ilorin, Ilorin, Nigeria

**Keywords:** genomic selection, factor analytic, preliminary yield trials, prediction accuracy, unstructured model, CDmean

## Abstract

To enable a scalable sparse testing genomic selection (GS) strategy at preliminary yield trials in the CIMMYT maize breeding program, optimal approaches to incorporate genotype by environment interaction (GEI) in genomic prediction models are explored. Two cross-validation schemes were evaluated: CV1, predicting the genetic merit of new bi-parental populations that have been evaluated in some environments and not others, and CV2, predicting the genetic merit of half of a bi-parental population that has been phenotyped in some environments and not others using the coefficient of determination (CDmean) to determine optimized subsets of a full-sib family to be evaluated in each environment. We report similar prediction accuracies in CV1 and CV2, however, CV2 has an intuitive appeal in that all bi-parental populations have representation across environments, allowing efficient use of information across environments. It is also ideal for building robust historical data because all individuals of a full-sib family have phenotypic data, albeit in different environments. Results show that grouping of environments according to similar growing/management conditions improved prediction accuracy and reduced computational requirements, providing a scalable, parsimonious approach to multi-environmental trials and GS in early testing stages. We further demonstrate that complementing the full-sib calibration set with optimized historical data results in improved prediction accuracy for the cross-validation schemes.

## Introduction

Due to climate change threatening crop productivity in sub-Saharan Africa (SSA), breeding for drought tolerance and yield stability across target environments is a high priority for the International Maize and Wheat Improvement Center (CIMMYT) tropical maize breeding program (Beyene et al., 2015, 2019). To achieve genetic gain improvement in alignment with these breeding objectives, the CIMMYT maize breeding programs leverage novel technologies such as doubled haploid (DH) technology, that allows generation of tens of thousands of inbred lines yearly, a low-cost genotyping platform, and genomic selection (GS) that uses whole-genome information to predict the genetic merit of new lines. The CIMMYT maize breeding scheme has five stages of testing. Many hybrid combinations are developed each year and tested in a small number of environments during the early testing phase, in later stages a small number of selected hybrid combinations are tested in many environments. To identify parental lines for the next breeding cycle and develop stress tolerant and high yielding hybrids that meet farmers’ needs, hybrids are tested under both well-watered (WW) and water-stress (WS) conditions in the preliminary screening stages. Each stage is characterized by the number of locations and the number of testers. These factors influence selection accuracy in the different testing stages.

At stage 1 or preliminary yield trials, several experimental hybrids are generated by crossing DH lines, or lines developed using the pedigree scheme, to a tester from a complementary heterotic group. The testcross hybrids are evaluated in 3–5 environments, where each environment is a combination of location and management (WS and WW), and the data are used to select the best 10–15 percent of the lines within or across the managements for advancement to stage 2 yield trials (Beyene et al., 2019). Effective selection decisions at stage 1 yield testing are critical for the advancement of lines with the greatest potential to perform in the resource-intensive multi-location, multi-tester testing stages. However, the effectiveness of phenotypic selection (PS) for stage 1 testcross trials is limited by evaluation on one tester and in few environments, which do not adequately represent the target population of environments (Endelman et al., 2014), this is largely due to the number of DH lines for testcross and the number of testcross hybrids for evaluation. Consequently, the CIMMYT Global Maize breeding program is focused on redesigning early-stage yield trials to accelerate genetic gain and reduce the cost of hybrid testing by evolving from a phenotypic based selection to the use of GS to predict the genetic merit of new lines. The efficiency of this method for evaluation of stage 1 candidates has been established (Beyene et al., 2019).

The current GS strategy relies on phenotyping 50 percent of a bi-parental population, observed across WW and WS environments, to predict the genetic merit of un-tested candidates for both WW and WS (Beyene et al., 2015, 2019; Santantonio et al., 2020) in a test-half-predict-half strategy (Atanda et al., 2020). While this strategy results in improved prediction accuracy at lower cost, it is not optimal for reducing breeding cycle time because a subset of the bi-parental population is required for model training (Atanda et al., 2020). The goal of the CIMMYT maize breeding program is to accelerate the early yield testing stage by using information from previously tested genotypes that have been phenotyped and genotyped (historical data) for model training. Based on the predicted genomic estimated breeding value (GEBV), lines will be advanced directly to stage 2 yield trials, the effectiveness of this strategy has been evaluated in our previous study.

Sparse testing represents a promising approach to expand the number of lines tested when GS is used to advance lines directly into stage 2, and for stage 1 screening of lines in cases where the genetic merit of some new lines may not be accurately predicted due to low genetic relationship between new lines and previously evaluated genotypes in the historical dataset. In the case where GEBV of lines cannot be accurately predicted from historical data, sparse testing has been identified as an optimal GS strategy compared to the current CIMMYT GS strategy (test-half-predict-half) that tests half of a full sib family to train genomic prediction models for full sibs that are not tested in stage 1 (Atanda et al., 2020; Santantonio et al., 2020). Given that all populations have phenotypic records in different environments, it is an appealing option for creating a robust historical dataset and allows for borrowing of information across environments resulting in improved prediction accuracy when compared to the test-half-predict-half strategy (Burgueño et al., 2012; Atanda et al., 2020; Santantonio et al., 2020).

To identify a scalable strategy that optimizes the representation of genetic space of the genotypes across environments leading to efficient use of information across the environments at the early yield testing stage, we evaluated two different breeding scenarios: (1) predicting the genetic merit of new bi-parental populations across environments (phenotyping of populations was unbalanced across environments) or, (2) predicting different subsets of a bi-parental population across environments. Here, coefficient of determination (CDmean) was used to split bi-parental populations across environments.

The main objectives of this study were to: (1) determine an effective strategy to implement sparse testing within the CIMMYT tropical maize breeding program and, (2) determine the optimal method to incorporate genotype by environment interaction (GEI) into the GS model for early yield testing stage.

## Materials and Methods

### Plant Materials

The datasets used in this study are described in detail in Atanda et al. (2020). Briefly, the maize datasets consist of 849 and 1,389 DH lines derived from 13 and 45 DH bi-parental populations respectively. The DH lines were unique within each year and were testcrossed to one of three single-cross testers in 2017 and one of two single-cross testers in 2018 respectively. Testcrosses in 2017 and 2018 were grouped into 13 and 34 trials, respectively. The trials were connected by common checks, and each trial was planted in an alpha-lattice incomplete block design with two replications under WW condition in Kiboko and Kakamega, Kenya and WS condition, in Kiboko during the 2017 and 2018 growing seasons. The entries in the trials were planted two-rows per plot, each row was 5 m long, with spacing of 0.75 m between rows and 0.25 m between hills. At planting, two seeds per hill were planted and thinned to one plant per hill 3 weeks after emergence to obtain a final plant population density of 53,333 plants per hectare. Fertilizers were applied at the rate of 60 kg N and 60 kg P_2_O_5_ per ha, as recommended for the area. Nitrogen was applied in a split dose at planting and 6 weeks after emergence. For the purposes of modeling genotype by environmental interactions (GEI), several combinations of factors (location, management, and year) were used to classify environments as summarized in Table 1.

**TABLE 1:** Classification of the environments based on management, location by management, management by year and location by management by year.

**Grouping of the environments**		**Environment**
Location by management	Kiboko by WW	LM1
	Kakamega by WW	LM2
	Kiboko by WS	LM3
Management (single year analysis)	WW	M1
	WS	M2
Management by year	WW by 2017	MY1
	WS by 2017	MY2
	WW by 2018	MY3
	WS by 2018	MY4
Management^++^ (multi-year analysis)	WW	M^+^1
	WS	M^+^2
Location by management by year	Kiboko by WW by 2017	LMY1
	Kakamega by WW by 2017	LMY2
	Kiboko by WS by 2017	LMY3
	Kiboko by WW by 2018	LMY4
	Kakamega by WW by 2018	LMY5
	Kiboko by WS by 2018	LMY6

*M^+^ is the broad classification of management across years as WW and WS.*

All DH lines were genotyped using repeat Amplification Sequencing (rAmpSeq) at Cornell Life Science Core Laboratory Center, Ithaca, NY, United States. The genotyping platform takes advantage of knowledge of whole-genome sequences and repetitive sequences to identify DNA sequence polymorphisms using novel bioinformatics tools [for detail see Buckler et al. (2016)]. It provides dominant markers, with the 9,155 sequence tags coded as 0 and 2 based on presence or absence of the dominant marker, respectively. The 6,785 markers with minor allele frequency greater than 0.05 were used for analysis.

### Genomic Selection Models

A separate analysis was run for each of the environmental classifications found in Table 1 using a multi-environment linear mixed model incorporating GEI effect. The covariance structures were defined using the groups in Table 1 and the model was fit in ASReml using the average information algorithm (Gilmour et al., 1995) as:

(1) $$y=1_{n}\,\mu +X_{1}\,b_{1}+Z_{1}\,u_{1}+Z_{2}\,u_{2}+Z_{3}\,u_{3}+Z_{4}\,u_{4}+Z_{5}\,u_{5}+\varepsilon$$

where y (n × 1) is the vector of phenotypes for each DH lines measured in the environments (1…k), μ is the overall mean and 1_n_ (n × 1) is a of vector ones, b_1_ is a fixed effect of location, u_1_is the random effect of the interaction between the genomic effect of g-th DH line and v-th environment, u_2_ is the random effect of the tester, u_3_is the random effect of the trial, u_4_ is the random effect of replication nested within environment, trial and year for the multi-year dataset, u_5_ is the random effects of incomplete block nested within replication, trial, location and year for the multi-year dataset. The number of fixed and random effects is represented as n and p, while X_n_ and Z_p_ are incidence matrices for fixed and random effects, respectively. The variance of the random effects u_2_, u_3_, u_4_, and u_5_were assumed to be distributed as:

(2) $${\text{u}}_{\text{p}}∼N\,\left(0,{\text{I}}_{\text{p}}\,\sigma_{{\text{u}}_{\text{p}}}^{2}\right)$$

where I_p_ and $\sigma_{{\text{u}}_{\text{p}}}^{2}$ are the identity matrix and variance of the p*-*th random effect (u_2_- u_5_). In Equation 1 all fixed effects and random effects u_2_- u_5_ are model in the same way for all analyses, while the covariance structure for u_2_ and ε varied based on the environmental classifications in Table 1.

The random GEI effect u_1_ is defined as the Kronecker product (⊗) between the g × g genomic relationship matrix (G) and the v × v variance-covariance matrix of the genomic effect of genotypes in and between environments (G_o_).

(3) $${\text{u}}_{1}∼N\,\left[0,\left(G⊗{\text{G}}_{\text{o}}\right)\right]$$

Thus, covariance of the genomic effect of the line (u_1_) in multi-environment model, can be represented as:

(4) $$C\,o\,v\,\left({\text{u}}_{1},{\text{u}}_{1}^{{}^{\prime }}\right)={\text{G}}_{\text{o}}⊗\text{G}$$

(5) $${\text{G}}_{\text{o}}⊗G=\left[\begin{array}{ccc} \sigma_{{\text{g}}_{1}}^{2} & \sigma_{{\text{g}}_{12}} & \begin{array}{cc} \cdots & \sigma_{{\text{g}}_{1\,v}} \end{array}\\ \sigma_{{\text{g}}_{21}} & \sigma_{{\text{g}}_{2}}^{2} & \begin{array}{cc} \cdots & \cdots \end{array}\\ \sigma_{{\text{g}}_{\mathrm{v1}}} & \begin{array}{c} \vdots \\ \vdots \end{array} & \begin{array}{cc} \begin{array}{c} \ddots \end{array} & \begin{array}{c} \vdots \\ \sigma_{{\text{g}}_{\text{v}}}^{2} \end{array} \end{array} \end{array}\right]⊗\text{G}\,\left(5\right)$$

where G_o_ represents the v × v variance-covariance matrix of the genomic effect of genotypes in the environments. The number of environments v varied based on the environmental classifications in Table 1. The diagonal of the G_o_ matrix is the additive genetic variance $\sigma_{{\text{g}}_{\text{v}}}^{2}$within the v-th environment. The off-diagonal (σ_g_1v_) elements represent the genetic covariance between environments.

Fitting the GEI in this way enables examination of the predictive ability of an unstructured model (US) that allows fitting unequal covariance between pairs of environments or managements, in addition to different genetic variances within environment/management. However, the number of parameters to estimate for the US model does not increase linearly with the number of environments, which can result in non-convergence when the number of model parameters is large relative to the number of data points (Smith et al., 2001; Kelly et al., 2007; Oakey et al., 2016). The factor analytic (FA) model has been identified as a more parsimonious approach to fit the complex covariance structure amongst a large number of environments (Piepho, 1998; Smith et al., 2001; Crossa et al., 2004; Oakey et al., 2016; Smith and Cullis, 2018). FA identifies one or few factors underlying the correlation among the k environments by their relationship to unobservable latent variables. Therefore, the GEI is modeled as interaction between the genomic effect of the g-th DH line and one or few factors underlying the environmental/management influences on the genotype (Piepho, 1998; Smith et al., 2001; Crossa et al., 2004; Kelly et al., 2007). FA model for Cov(${\text{u}}_{\text{g}},{\text{u}}_{\text{g}}^{{}^{\prime }}$) is expressed as:

(6) $$\left(\Lambda \,\Lambda^{\prime }+\Psi \right)⊗G$$

where Λ is a v × m matrix of loading factors, the columns of Λ are associated with the environmental loadings for the m-th latent factor. Ψ is a v × v heterogeneous diagonal matrix with specific environment genetic variances Ψ_v_ on the diagonal and zero covariance between environments. When the number of environments was less than 4 (as defined in Table 1), one multiplicative component was considered (m = 1) and m = 2 as number of environments increased from 4 to 6. We use the extended FA (XFA) model that allows a non-full rank variance matrix for the GEI effects, therefore the mixed model equation is sparser, resulting in reduced computational requirements compared to the standard FA model. Details can be found in Thompson et al. (2003) and Meyer (2009).

The residual variance for the GS model (Equation 1) can be specified as:

(7) ε∼N⁢(0,R)

where R is a heterogeneous diagonal matrix of the residual variances for each environment v:

(8) $$R=\left[\begin{array}{ccc} \sigma_{\varepsilon_{1}}^{2}*{\text{I}}_{n_{1}} & 0 & \begin{array}{cc} \cdots & 0 \end{array}\\ 0 & \sigma_{\varepsilon_{2}}^{2}*{\text{I}}_{n_{2}} & \begin{array}{cc} \cdots & 0 \end{array}\\ \begin{array}{c} \vdots \\ 0 \end{array} & \begin{array}{c} \vdots \\ 0 \end{array} & \begin{array}{cc} \begin{array}{c} \ddots \end{array} & \begin{array}{c} \vdots \\ \sigma_{\varepsilon_{\text{v}}}^{2}*{\text{I}}_{\text{n\_v}} \end{array} \end{array} \end{array}\right]$$

where I_n_v__ is a n_v_ = n_v_ identity matrix and n_v is the number of observations in environment v. The off-diagonal elements of the R matrix equal zero [Cov(ε,ε′) = 0] and diagonal elements represent the residual variance within each of v environments. Generally, the residual variance for multi-environment GS models can take two different forms explaining different model assumptions. For example, a uniform residual variance for all environments ($\sigma_{\varepsilon_{1}}^{2}$ = $\sigma_{\varepsilon_{2}}^{2}\,\ldots$ = $\sigma_{\varepsilon_{\text{v}}}^{2}$), and a heterogeneous residual variance where each environment has different residual variance ($\sigma_{\varepsilon_{1}}^{2}\neq \sigma_{\varepsilon_{2}}^{2}\,\ldots \neq \sigma_{\varepsilon {}_{\text{v}}}^{2}$).

The plot level heritability for each environment was calculated from the variance components obtained from the model as:

(9) $${\text{h}}_{\text{v}}^{2}=\frac{\sigma_{{\text{g}}_{\text{v}}}^{2}}{\sigma_{{\text{g}}_{\text{v}}}^{2}+\sigma_{\varepsilon_{\text{v}}}^{2}}$$

where $\sigma_{{\text{g}}_{\text{v}}}^{2}$ and $\sigma_{\varepsilon_{\text{v}}}^{2}$ are the genetic and residual variance estimates specific to environment v.

### Calibration Set Optimization Criteria

Following Atanda et al. (2020), CDmean and Avg_GRM were used as genetic optimization criteria. Similar to Rincent et al. (2012), CDmean was used to optimize experimental design by determining which individuals were evaluated in each environment. However, in this study, CDmean is the mean of the expected reliability of the predicted genetic values of N-1 individuals in a specific bi-parental population, where N is the size of a given full-sib family with each g-th individual used to predict the reliability of the remaining full-sibs. The expected reliability of the prediction of the different contrasts was expressed as:

(10) $$\text{CD}\,\left(\text{K}\right)=\mathrm{diag}\,\left[\frac{{\text{K}}^{{}^{\prime }}\,\left(G-\lambda \,{\left({\text{Z}}^{{}^{\prime }}\,\text{DZ}+\lambda \,{\text{G}}^{-1}\right)}^{-1}\right)\,K}{{\text{K}}^{{}^{\prime }}\,\text{GK}}\right]$$

where D = 1−X(X′X)−1X′, G, X, and Z are the same as defined above and K is a matrix of contrast vectors with the sum of each contrast vector equal to zero such that 1′K = 0.

In principle λ = $\sigma_{\varepsilon }^{2}/\sigma_{\text{g}}^{2}$, where $\sigma_{\varepsilon }^{2}$ is the residual error and $\sigma_{\text{g}}^{2}$ is the genetic variance obtained from Equation 1; however, this cannot be calculated for untested lines. According to Atanda et al. (2020), the efficiency of CDmean is not highly dependent on trait heritability but rather on genomic relationship. Consequently, λ was set to 0.5. In our previous study, when an intermediate value was chosen for (λ = 0.5) the prediction accuracy was close to accuracies achieved using λ = $\sigma_{\varepsilon }^{2}/\sigma_{\text{g}}^{2}$, this was in agreement with Rincent et al. (2012). Therefore, CDmean = mean [diag(CD(K))], each column of the K matrix is a contrast between (N-1) individuals of a full-sib family and the mean of the full-sib family. A contrast using the first individual in the family is set up as:

(11) $${\text{K}}_{1}=\text{c}\left(\frac{n-1}{\text{n}},\frac{-1}{\text{n}},\frac{-1}{\text{n}}\right)$$

Where n is the number of individuals in the populations. Therefore, one individual of a full-sib in a specific bi-parental population serves as a calibration set to estimate the reliability of predicting the remaining full-sibs. This was repeated N times enabling each g-th individual of a full-sib to serve as calibration set. Consequently, we obtain a CDmean value for each individual in a given bi-parental population and individuals (50 percent of a bi-parental population) with the highest CDmean value represent an optimized calibration set. Theoretically, individuals with high CDmean value maximize the reliability of those with low CDmean value, thus full-sib families where split between environment by keeping high and low CDmean lines together in WW environments, respectively. In the WS environment, a portion of lines from each WW environment were used as the calibration set (Supplementary Figure 2). A script to calculate the CDmean is provided in Supplementary File 1. This strategy was adopted because it is computationally efficient compared to Rincent et al. (2012) which used an exchange algorithm to randomly exchange one individual between the calibration set (N′, – total number of individuals to phenotype) and the un-phenotyped individuals (N-N′), where the exchange is accepted if the initial CDmean value improved and rejected otherwise. The process repeated until reaching a plateau. Akdemir et al. (2015) and Heslot and Feoktistov (2020) also modified Rincent et al. (2012) with improved computational efficiency. The efficacy of these methods was not compared in our study, but results from preliminary analysis show the strategy used in this study improved prediction accuracy compared to Rincent et al. (2012) (results not shown).

The Avg_GRM is a raw estimate of the proportion of the genome shared between a potential training set and all individuals in a specific full-sib family. Based on the results from our previous study (Atanda et al., 2020), CDmean and Avg_GRM genetic optimization criteria have similar efficiency in selecting individuals from historical data closely related with a specific bi-parental population. However, Avg_GRM genetic optimization criterion is computationally more efficient; thus, the Avg_GRM genetic optimization criterion was used to select 300 individuals from the historical data that are closely related to a specific full-sib family. The Avg_GRM can be expressed as:

(12) $${\text{Avg\_GRM}}_{\text{j}}=\frac{1}{\text{n}}\,\sum_{\text{g}}^{\text{n}}{\text{G}}_{\text{gj}}$$

where G_gj_ is the genomic relationship between the g-th individual in a target full-sib family and the j-th line in the historical data and n is the size of target full-sib family.

### Cross-Validation Scheme

The predictive ability of two cross-validation schemes was evaluated for possible implementation of a sparse testing GS strategy in the CIMMYT tropical maize breeding program. For even distribution of populations across environments, a bi-parental population with size ≤ 30 was dropped from 2017 dataset and the remaining 12 bi-parental populations were used for the analysis. The first cross-validation scheme (CV1) involved masking six random bi-parental populations of the twelve bi-parental populations in one WW environment with the remaining bi-parental populations masked in the other WW environment. In the WS environment, three random bi-parental populations from each WW environment were masked; this process was repeated 10 times (Supplementary Figure 1). Prediction accuracy was calculated as the Pearson correlation of the predicted GEBV obtained from the models and the BLUE estimates of DH testcrosses for each population in each environment. The mean across populations is reported.

**FIGURE 1:**
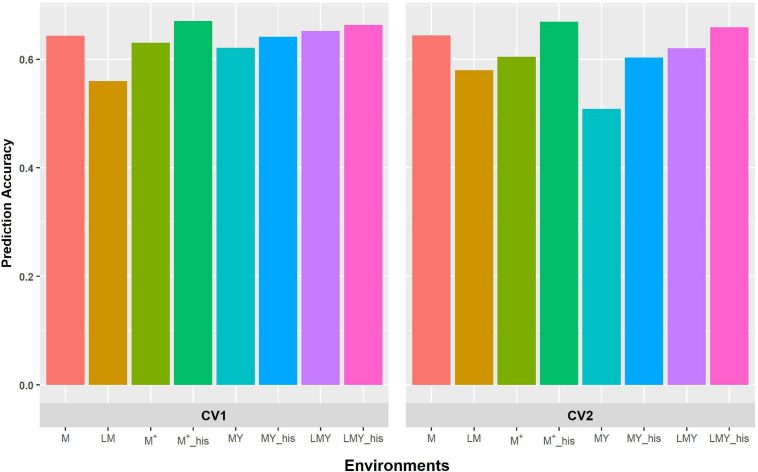
Predictive ability of factor analytic model for the cross-validation schemes (CV1 and CV2) in WS environments/management. LM and M represent prediction accuracy obtained when covariance was modeled across environments and managements, respectively, for within-year prediction. LMY represents classification of environment as location by management by year, MYand M ^+^ represent the broad classification of the management across years as WW and WS, and explicit definition of the management across years as WW 2017 and 2018 and WS 2017 and 2018. LMY, MY and M^+^ used all available historical data. The suffix “his” represents prediction accuracy obtained with optimized historical data using the Avg_GRM genetic optimization criterion.

In the second cross-validation scheme (CV2), CDmean was used for splitting each bi-parental population equally across WW environments by masking 50 percent of a bi-parental population with lowest CDmean value in one environment and the remaining 50 percent masked in the other WW environment. For the WS environment, half of the individuals unmasked in the WW environments were masked (Supplementary Table 1 and Figure 1). Due to the diversity of populations in 2018, the 2018 dataset was chosen to represent “historical” data in this study. Following Atanda et al. (2020), we further assessed the predictive ability of augmenting the training set in both cross-validation schemes with all historical data or with an optimized set of 300 individuals from the historical records closely related to a specific full-sib family using Avg_GRM genetic optimization criterion. In the scenario where full-sib training sets were augmented with historical data, GEI was considered as location by management by year (LMY 1, 2, 3, 4, 5, and 6), management by year (MY 1, 2, 3, and 4) to account for the difference between managements across years in addition to the broad definition of management as WW (M ^+^ 1) and WS (M ^+^ 2). The prediction accuracy was calculated as the Pearson correlation of the predicted GEBV and the BLUE estimates of DH lines in each environment, obtained using the complete dataset for each population, from the combined analysis. The mean across populations is reported.

## Results

### Residual Variance, Heritability Within Environment/Management, and Correlation Between Pairs of Environments/Managements

Except for when the environment was classified as year by management by location (LMY 1, 2, 3, 4, 5, and 6), where the US model was responsive to the training set and did not consistently converge, the results for FA and US models were equivalent regardless of the cross-validation schemes (Result not shown). Thus, only results from FA model were presented. The genetic correlation between environments (LM 1, 2, and 3) in the CV1 ranges from 0.13 to 0.64 (Table 2). A similar trend was observed for CV2 and ranges from 0.22 to 0.363. For CV1, the within environments (LM 1, 2, and 3) plot-level heritability for grain yield ranges from 0.27 to 0.42 and ranges from 0.26 to 0.32 in CV2. When environments were grouped into managements, for CV1, the genetic correlation between WW (M1) and WS (M2) was 0.37 and plot-level heritability within each management was 0.24 and 0.35 respectively. While for CV2, the genetic correlation between M1 and M2 was 0.47, and plot-level heritability within each management was 0.19 and 0.32.

**TABLE 2:** Plot level heritability (diagonal) and genetic correlations between pairs of managements or environments (upper diagonal) for the two managements (upper half) and three environments (lower half) from the factor analytic model analysis of 2017 dataset.

	Cross-validation scheme					
		**CV1**			**CV2**	
M	WW	WS		WW	WS	
WW	0.24 (0.08)	0.37	–	0.19 (0.06)	0.47	–
WS		0.35 (0.06)	–		0.32 (0.09)	–
LM	Kiboko WW	Kakamega WW	Kiboko WS	Kiboko WW	Kakamega WW	Kiboko WS
Kiboko WW	0.27 (0.09)	0.24	0.63	0.26 (0.06)	0.31	0.63
Kakamega WW		0.42 (0.06)	0.15		0.32 (0.10)	0.22
Kiboko WS			0.34 (0.06)			0.32 (0.04)

*M represents grouping of locations by management as WW and WS; LM represents the grouping of locations as Kiboko-WW, Kakamega-WW and Kiboko-WS. Plot level heritability estimates within each grouping management (M or LM) are represented in the diagonal. The upper diagonals are genetic correlations between environmental groupings. Standard errors for the heritability estimates are in parentheses.*

The genetic correlation between environments (LMY 1, 2, 3, 4, 5, and 6) varies across the cross-validation schemes, it ranges from −0.14 to 0.74 for CV1 and −0.02 to 0.79 for CV2. The plot level heritability for each environment across the cross-validation was modest. In analyses where management was defined across years (WW 2017 and 2018 – MY1 and 3, WS 2017 and 2018 – MY2 and 4), the genetic correlation between managements also ranged from negative to moderate correlation for CV1 (Table 3). While it ranged from low to moderate in CV2. For the broad definition of management across years as WW (M ^+^ 1) and WS (M ^+^ 2), the genetic correlation was 0.60 and 0.68 for CV1 and CV2, respectively. Generally, the estimates of plot-level heritability for CV1 and CV2 were moderate.

**TABLE 3:** Plot level heritability (diagonal) and genetic correlations between pairs of managements (upper diagonal) for the two managements (upper half) and four managements (lower half) from the factor analytic model analysis of combined 2017 and 2018 dataset.

	**CV1**					
M^+^	WW	WS				
WW	0.31 (0.05)	0.60	–	–	–	–
WS		0.38 (0.03)	–	–	–	–
MY	WW 2017	WS 2017	WW 2018	WS 2018		
WW 2017	0.32 (0.03)	0.31	0.10	0.05	–	–
WS 2017		0.38 (0.03)	−0.11	0.55	–	–
WW 2018			0.27 (0.05)	0.09	–	–
WS 2018				0.20 (0.03)	–	–
LMY	Kiboko WW 2017	Kakamega WW 2017	Kiboko WS 2017	Kiboko WW 2018	KakamegaWW 2018	Kiboko WS 2018
Kiboko WW 2017	0.30 (0.07)	−0.03	0.45	0.04	−0.14	0.19
Kakamega WW 2017		0.46 (0.08)	−0.10	0.29	0.38	0.16
Kiboko WS 2017			0.41 (0.04)	0.23	−0.10	0.32
Kiboko WW 2018				0.49 (0.04)	0.69	0.74
KakamegaWW 2018					0.50 (0.08)	0.33
Kiboko WS 2018						0.38 (0.04)

			**CV2**			

M^+^	WW	WS				
WW	0.35 (0.04)	0.68				
WS		0.39 (0.05)				
MY	WW 2017	WS 2017	WW 2018	WS 2018		
WW 2017	0.35 (0.04)	0.47	0.36	0.20		
WS 2017		0.38 (0.04)	0.30	0.59		
WW 2018			0.15 (0.07)	0.38		
WS 2018				0.20 (0.05)		
LMY	Kiboko WW 2017	Kakamega WW 2017	Kiboko WS 2017	Kiboko WW 2018	KakamegaWW 2018	Kiboko WS 2018
Kiboko WW 2017	0.27 (0.07)	−0.01	0.32	0.12	−0.10	0.19
Kakamega WW 2017		0.38 (0.05)	0.38	0.42	0.54	0.12
Kiboko WS 2017			0.38 (0.06)	0.26	−0.02	0.34
Kiboko WW 2018				0.53 (0.10)	0.73	0.79
KakamegaWW 2018					0.54 (0.05)	0.55
Kiboko WS 2018						0.36 (0.05)

*M ^+^ represents broad classification of management across years as WW and WS. MY represents the grouping of environments by management (WW and WS) and year (2017 and 2018). LMY groups environments by management (WW and WS), location (Kakamega and Kiboko), and year (2017 and 2018). Plot level heritability estimates for M ^+^, MY, and LMY are represented in the diagonal. The upper diagonals are genetic correlations between environmental groupings. Standard errors for the heritability estimates are in parentheses.*

### Comparison of Predictive Ability of the Models and the Cross-Validation Schemes

**FIGURE 2:**
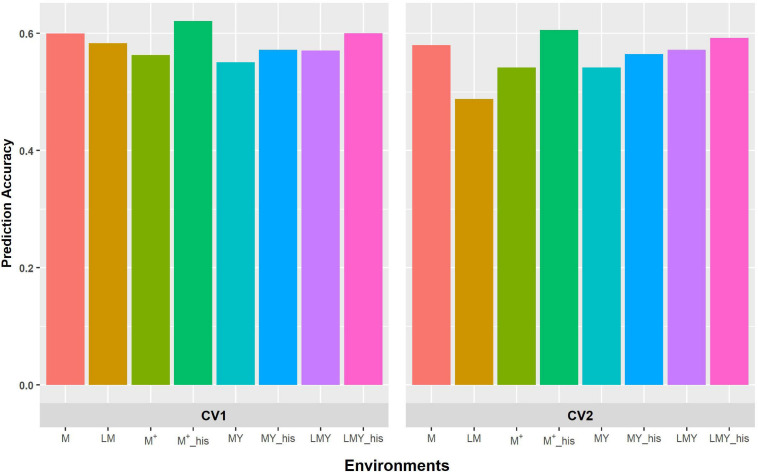
Predictive ability of the factor analytic model for the cross-validation schemes (CV1 and CV2) in WW environments/management. LM and M represent prediction accuracy obtained when covariance was modeled across environments and managements, respectively, for within-year prediction. LMY represents classification of environment as location by management by year, MY and M ^+^ represent the broad classification of the management across years as WW and WS, and explicit definition of the management across years as WW 2017 and 2018 and WS 2017 and 2018. LMY, MY and M^+^ used all available historical data. The suffix “his” represents prediction accuracy obtained with optimized historical data using the Avg_GRM genetic optimization criterion.

The grouping of the environments into management consistently shows higher prediction accuracy compared to modeling of covariance between environments defined as a combination of location, management and year (Figures 1, 2). Though the prediction accuracy for the cross-validation schemes was similar, the slight difference corroborates the different estimates of heritability and genetic correlation obtained from the cross-validation schemes. The augmentation of the training set with optimized historical information improved prediction accuracy compared to either use of all the historical data plus the full-sib training set or only the full-sib training set. Unsurprisingly, prediction accuracy increases with higher heritability and genetic correlation between environments/managements as observed with prediction accuracy of WW compared to WS. Although prediction accuracy of FA and US models are similar (Supplementary Table 2), the US model failed to consistently converge when environment was defined based on the combination of location, management, and year.

## Discussion

The sparse testing GS strategy in which the genetic merit of new lines is evaluated in different but genetically correlated environments has proven to increase prediction accuracy compared to the test-half-predict-half GS strategy and, provided that all new lines have phenotypic data, it is seemingly robust for developing historical training datasets (Burgueño et al., 2012; Atanda et al., 2020; Santantonio et al., 2020). The evaluation of new genotypes across environments allows the utilization of information across environments using multi-environment models. However, multi-environment models, especially the US model, tend to become non-parsimonious as the number of environments increases resulting in convergence failure (Smith et al., 2001; Kelly et al., 2007; Meyer, 2009). Considering that a small number of environments and genotypes were evaluated in the preliminary yield trials in this study, the use of the US model did not pose any statistical challenge. However, inclusion of historical data in the training set increases the number of environments, which could result in computational challenges for the US approach. Alternatively, the FA model, which is a complexity reduction model for an increased number of environments, requires fewer parameters while accounting for covariance between environments (Smith et al., 2001; Thompson et al., 2003; Crossa et al., 2004; Kelly et al., 2007; Burgueño et al., 2008, 2011, 2012; Smith and Cullis, 2018; Tolhurst et al., 2019), and could be more suitable as historic training datasets increase in size and complexity.

Although the predictive ability of the two cross-validation schemes is comparable, the improved prediction accuracy of CV1 might be due to the close relationship (half-sib relationship) of all the populations. Previous studies (Lehermeier et al., 2014; Schopp et al., 2017; Atanda et al., 2020) also indicate that use of closely related multiple bi-parental populations as a training set result in improved prediction accuracy. Using diverse populations, one would expect the differences in marker-quantitative trait loci linkage phase across bi-parental populations would result in a lower signal to noise ratio, but that does not appear to be the case in this dataset where several populations share a common parent. The small size of the bi-parental population used in this study might affect the prediction accuracy of CV2. Borrowing of information across environments was the basis for the improved prediction accuracy using sparse testing compared to test-half-predict-half (Atanda et al., 2020), thus, a strategy that optimizes coverage of the genetic space of the genotypes across environments should result in higher predictive ability.

The FA is a parsimonious model for fitting a relatively high number of environments in multi-environment trials utilizing latent factors which give rise to correlations between environments to capture the complexity of covariances among many environments (Burgueño et al., 2012; Oakey et al., 2016; Smith and Cullis, 2018; Tolhurst et al., 2019). However, with few environments and a large dataset to estimate all model parameters, the superiority of the FA model over the US model will likely depend on the ability of the FA model to adequately represent the underlying covariance structure between environments in the dataset (Piepho, 1998; Kelly et al., 2007; Meyer, 2009; So and Edwards, 2009; Ward et al., 2019). While this study looked at relatively few environments, the limitations of the US model became apparent in the multi-year dataset with six environments defined. Under this scenario, US model was sensitive to the training set used and did not consistently converge, suggesting that the utility of US model will diminish rapidly as the number of environments increase. Given reliable convergence and similar performance with a small number of environments, the FA appears to be a more robust approach for modeling sparse testing implementations in the CIMMYT Maize program.

In practice, the CIMMYT tropical maize breeding program advances lines to multi-location, multi-tester yield trials based on relative performance within or across managements (WW and WS), the observed improvement in prediction accuracy when environments were grouped into managements suggests that categorizing the environments into management did not sacrifice information on GEI. Assigning environments/locations into groups using prior information, such as management, as is the case in this study, can serve as a complexity reduction strategy for reducing the number of model parameters, providing a more parsimonious approach for modeling GEI. However, stage 1 yield testing is typified by a small number of environments, which is a limitation to the generalization of the results of this study across different phases of yield testing, in particular with a large number of environments. However, similar to the strategy employed in this study, using multi-environment data, Lado et al. (2016) grouped 35 environments into three mega environments using the additive main and multiplicative interactive (AMMI) model (Zobel et al., 1988), and GS was performed within the mega environments.

Augmenting a given full-sib training set with an optimized set of 300 individuals from historical data using the Avg_GRM genetic optimization algorithm improved prediction accuracy compared to using all available historical records. The similar genetic covariance between managements, heritability, and prediction accuracy obtained when historical data is used to complement the full-sib training set, suggests that an increase in the training set size using historical data results in more stable estimates of model parameters when compared to using only the full-sib records as the training set. The results from this study corroborate our earlier study (Atanda et al., 2020) indicating that the use of genetic optimization criteria to select individuals genetically connected to the breeding population to serve as a training population results in improved prediction accuracy. This further illustrates the importance of genetic relationships between training and breeding populations and indicates that any GS approach carefully consider which historical records are included for training of genomic prediction models. Furthermore, these results suggest that, when genomic information is available breeders should consider utilizing multi-year information for advancement decisions. This could not only improve advancement decisions but could enable earlier recycling of material to reduce generation intervals.

## Conclusion

Given the similar prediction accuracies obtained in CV1 and CV2, decisions on which sparse testing experimental design will likely depend on cost and ease of implementation. While the prediction accuracy for the cross-validation schemes is equivalent, CV2 has an intuitive appeal in that all bi-parental populations have representation across environments, which would allow efficient use of information across environments and would be ideal for building a robust historical dataset. Further, the CV2 can be extended to resource demanding multi-environment, multi-tester advanced yield testing stages to save resources. In this study, grouping similar environments to model GEI information reduced computational challenges and achieved superior prediction accuracy. In general, including historical information in trial advancement decisions improved prediction accuracy, suggesting that the use of historical information in routine advancement decisions could improve accuracy. Furthermore, selecting historical information based on genetic connectedness with the breeding population proved more effective than including all historical information.

## Data Availability Statement

The original contributions presented in the study are included in the article/Supplementary Material, further inquiries can be directed to the corresponding author/s.

## Author Contributions

MO, KR, and SA conceptualized the study. SA analyzed, interpreted the result, and drafted the manuscript. YB coordinated the field experiments. MG and KD were responsible for phenotypic and genotyping data management. JB, JC, RR, DD, PB, PT, ED, GO, and other authors contributed to the editing of the manuscript. All authors contributed to the article and approved the submitted version.

## Conflict of Interest

The authors declare that the research was conducted in the absence of any commercial or financial relationships that could be construed as a potential conflict of interest. The handling editor declared a past co-authorship with several of the authors JC, YB, MG, JB, and PB.

## Supplementary Material

Supplementary Figure 1 Illustration of cross-validation scheme 1. Each box represents a population; the black color depicts populations masked in an environment and the white color represents populations used for model training to predict the genomic estimated breeding value of masked populations in each environment. Environments (1, 2, and 3) represent Kiboko optimal, Kakamega optimal and Kiboko drought.

Supplementary Figure 2 Illustration of cross-validation scheme 2. Each box represents a population; the white color depicts individuals within a bi-parental population selected based on their CDmean value to predict the genomic estimated breeding value of masked individuals (black color). Environments (1, 2, and 3) represent Kiboko optimal, Kakamega optimal and Kiboko drought.

Supplementary Table 1 Masking of subset of a bi-parental population in CV2 across environments.

Supplementary Table 2 Prediction accuracy for factor analytic models using *m* = 1 and 2 and the unstructured model, depicting model accuracy using either *m* = 1 or 2 as number of environments increase.

Supplementary Table 3 Eigen analysis of factor analytic matrix, showing variation explained by the latent variables.
