# Supplementary figures and images for: Scalable Sparse Testing Genomic Selection Strategy for Early Yield Testing Stage
Source: Front Plant Sci. 2021 Jun 22;12:658978. doi: 10.3389/fpls.2021.658978 (PMC8259603; doi:10.3389/fpls.2021.658978)

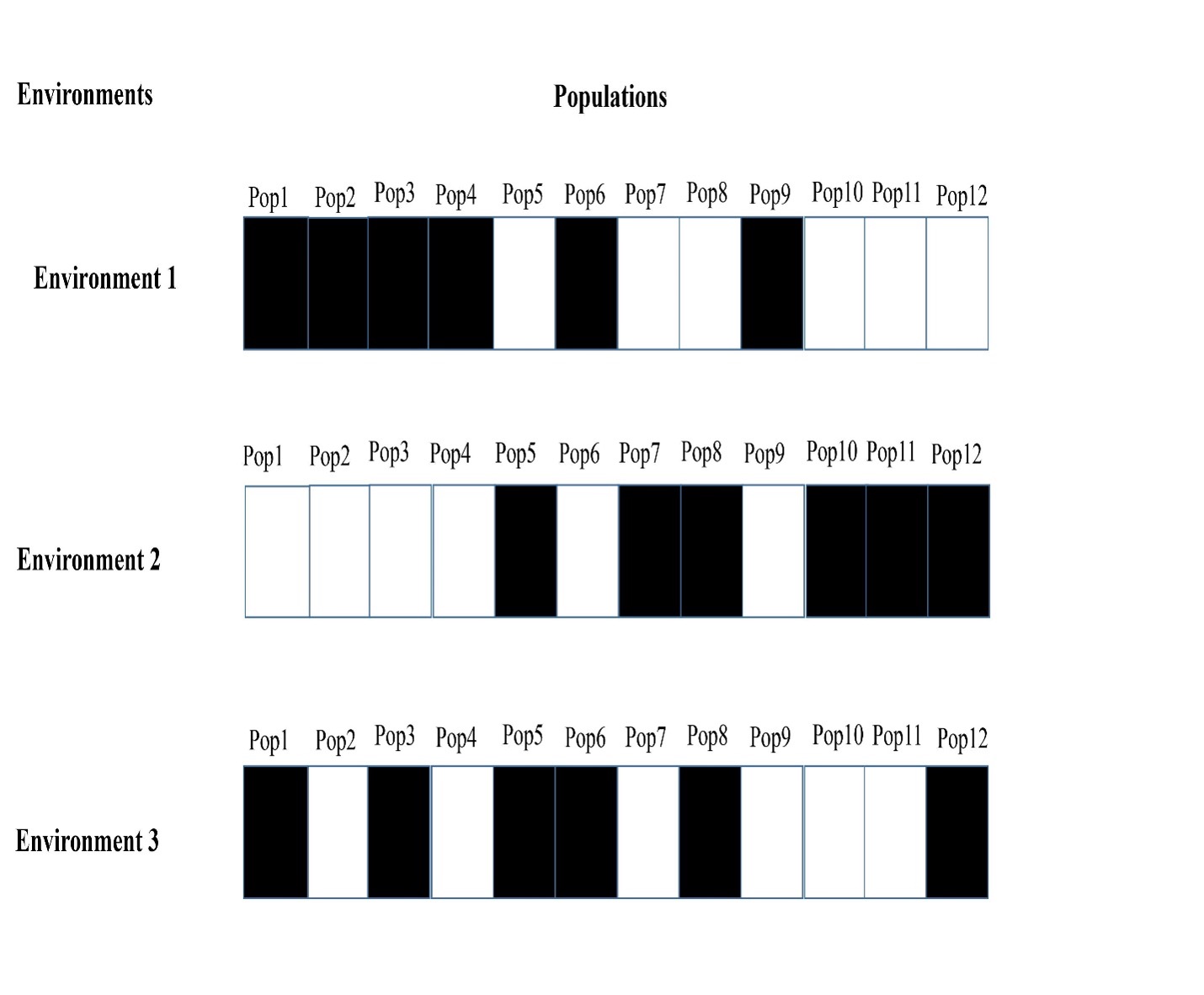

Supplement: Supplementary Figure 1 — Illustration of cross-validation scheme 1. Each box represents a population; the black color depicts populations masked in an environment and the white color represents populations used for model training to predict the genomic estimated breeding value of masked populations in each environment. Environments (1, 2, and 3) represent Kiboko optimal, Kakamega optimal and Kiboko drought. [file Image_1.JPEG]

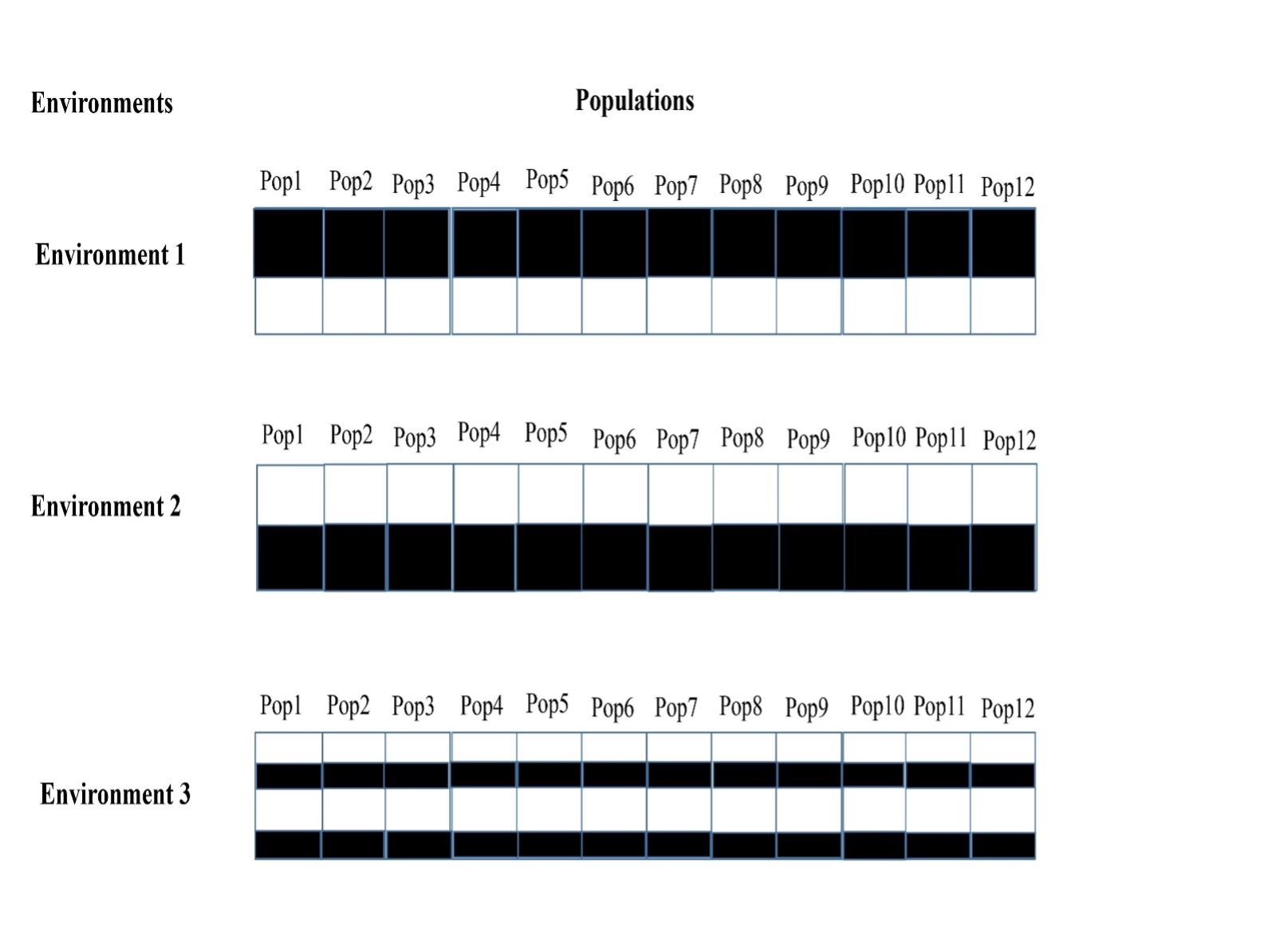

Supplement: Supplementary Figure 2 — Illustration of cross-validation scheme 2. Each box represents a population; the white color depicts individuals within a bi-parental population selected based on their CDmean value to predict the genomic estimated breeding value of masked individuals (black color). Environments (1, 2, and 3) represent Kiboko optimal, Kakamega optimal and Kiboko drought. [file Image_2.JPEG]
