# Supplementary material for: Scalable Sparse Testing Genomic Selection Strategy for Early Yield Testing Stage
Source: Front Plant Sci. 2021 Jun 22;12:658978. doi: 10.3389/fpls.2021.658978 (PMC8259603; doi:10.3389/fpls.2021.658978)
Supplement: Supplementary Table 1 — Masking of subset of a bi-parental population in CV2 across environments. [file Data_Sheet_2.docx]

Supplementary Table 1: Masking of subset of a bi-parental population in CV2 across environments

|  |  | **Optimal** | | **Drought** |
| --- | --- | --- | --- | --- |
| **Populations** | **Size** | **Kiboko** | **Kakamega** | **Kiboko Drought** |
| **POP2** | 87 | 43 | 44 | 44 |
| **POP3** | 83 | 41 | 42 | 42 |
| **POP4** | 91 | 46 | 45 | 46 |
| **POP5** | 108 | 54 | 54 | 54 |
| **POP6** | 68 | 34 | 34 | 34 |
| **POP7** | 44 | 22 | 22 | 22 |
| **POP8** | 88 | 44 | 44 | 44 |
| **POP9** | 53 | 26 | 27 | 27 |
| **POP10** | 37 | 18 | 19 | 19 |
| **POP11** | 31 | 16 | 15 | 16 |
| **POP12** | 66 | 33 | 33 | 33 |
| **POP13** | 63 | 31 | 32 | 32 |

Size is the total number of individuals in each bi-parental population used in this study. The columns for each environment show the number of full-sibs within a bi-parental population that had phenotypes masked.

Supplementary Table 2: Prediction accuracy for factor analytic models using m =1 and 2 and the unstructured model, depicting model accuracy using either m =1 or 2 as number of environments increase.

|  |  |  | Optimal | | Drought | |
| --- | --- | --- | --- | --- | --- | --- |
|  |  | Number of parameters | CV1 | CV2 | CV1 | CV2 |
| Env = 2 | XFA1 | 4 | 0.64 | 0.64 | 0.59 | 0.58 |
|  | US | 3 | 0.65 | 0.64 | 0.61 | 0.58 |
|  |  |  |  |  |  |  |
| Env = 3 | XFA1 | 6 | 0.56 | 0.58 | 0.58 | 0.49 |
|  | US | 6 | 0.57 | 0.56 | 0.55 | 0.44 |
|  |  |  |  |  |  |  |
| Env = 4 | XFA1 | 8 | 0.59 | 0.49 | 0.55 | 0.35 |
|  | XFA2 | 11 | 0.62 | 0.51 | 0.57 | 0.55 |
|  | US | 10 | 0.62 | 0.51 | 0.57 | 0.55 |
|  |  |  |  |  |  |  |
| Env = 6 | XFA1 | 12 | 0.63 | 0.60 | 0.53 | 0.49 |
|  | XFA2 | 17 | 0.66 | 0.62 | 0.57 | 0.57 |

For the CV1, accuracy was calculated by correlating the GEBV estimates of the masked populations (six) in each environment with the BLUE estimates for each environment using the complete dataset. For the CV2 phenotypes for 50% of the lines from each population were masked in each environment based on CDmean groupings, and accuracy was calculated by correlating the GEBV of masked lines with the BLUE estimates for each environment estimated using the complete dataset. The mean across populations is reported for the each cross-validation scheme.

Supplementary Table 3: Eigen analysis of factor analytic matrix, showing variation explained by the latent variables.

| ENV = 2 | Eigen Value | 0.48 |  | 0.15 |  |  |  |  |
| --- | --- | --- | --- | --- | --- | --- | --- | --- |
|  | Percentage | 76.36 |  | 23.64 |  |  |  |  |
| ENV= 3 | Eigen Value | 1.29 |  | 0.54 | 0.14 |  |  |  |
|  | Percentage | 65.33 |  | 27.44 | 7.23 |  |  |  |
| ENV = 4 | Eigen Value | 0.89 |  | 0.75 | 0.28 | 0.19 |  |  |
|  | Percentage | 41.89 |  | 35.27 | 13.66 | 9.17 |  |  |
| ENV = 6 | Eigen Value | 3.48 |  | 1.39 | 0.59 | 0.55 | 0.26 | 0.16 |
|  | Percentage | 54.10 |  | 21.57 | 9.25 | 8.52 | 4.02 | 2.55 |
