# Supplementary material for: Scalable Sparse Testing Genomic Selection Strategy for Early Yield Testing Stage
Source: Front Plant Sci. 2021 Jun 22;12:658978. doi: 10.3389/fpls.2021.658978 (PMC8259603; doi:10.3389/fpls.2021.658978)
Supplement: Supplementary file 4 [file Data_Sheet_1.PDF]

Scalable Sparse Testing Genomic Selection Strategy for Early Yield Testing Stage

Supporting R code

Sikiru Adeniyi Atanda, Michael Olsen, Jose Crossa et al.

Contents

1 Load Packages 2
2 Load data 2
3 Function to CDmean value for each full-sibs in a specific specific bi-parental population 2

This document illustrates how the CDmean explained in Rincent et al. (2012) in Genetics 192(2):715-728, doi: 10.1534/genetics.112.141473 was modified to estimate CDmean of individual full-sibs in a full-sib family. To cover the genetic space across environments, the full-sibs were split across environments based on CDmean value.

1 Load Packages

```
packages <- c("data.table", "Matrix", "matrixcalc", "MASS")

libraries <- function(packages){

      for(package in packages){
         #checks if package is installed
         if(!require(package, character.only = TRUE)){
            #If package does not exist, then it will install
            install.packages(package, dependencies = TRUE)
            #Loads package
            library(package, character.only = TRUE)
         }
      }
   }


libraries(packages)
```

2 Load data

```
# Import Reference File containing both the populations and
# the GID of individuals in the population

# Referenec must contain two column, one represent population class
# and GID of the genotypes

ref = read.csv("", header = T, as.is = T, sep = ",", stringsAsFactors = F)



### The Kinship matrix

Kin <-  read.csv("", header = T, as.is = T, sep = ",", stringsAsFactors = F, row.names = 1)


# Vector of all bi-parental populations
POPs = c()
```

3 Function to CDmean value for each full-sibs in a specific specific bi-parental population

The function computes the CDmean value for for all full-sibs in a specific bi-parental population for all populations

```
TRN.Opt.Algo <- function(Reference, Gmatrix, POPs, percent) {
```

```r
#percent: the percentage of splitting the population
#POPs_TRN: list of all population
#Output_E1 : Optimized training set in Environment 1
#Output_E2 : Optimized training set in Environment 2
#Output_E3 : Optimized training set in Environment 3



if (!is.null(POPs)) {
POPs_TRN = vector("list", length = length(POPs))

Output_E1 = vector("list", length = length(POPs))

Output_E2 = vector("list", length = length(POPs))

Output_E3 = vector("list", length = length(POPs))


         } else {

         stop()

         }


for (s in 1:length(POPs)) {
Reference2 =  Reference[Reference[, 1] %in% POPs[s], ]

if (!is.character(Reference2[, 2]))
Reference2 <- as.character(Reference2[, 2])
if (is.unsorted(Reference2[, 2]) == T)
Reference2 <- Reference2[order(Reference2[, 2]), ]


# Vector of Individuals in the Validation set
Inds_in_a_POP =  Reference2[Reference2[, 1] %in% POPs[s], 2]

NTrnSample = ceiling(length(Inds_in_a_POP) * percent)


# Create dataframe to store the obtained CDmean value for for all full-sibs
# in a specific bi-parental population

CDmean = data.frame(CDmean = rep(NA, length(Inds_in_a_POP)), GID = Inds_in_a_POP)


# Create the list of all the full-sibs that will be used as validation set for each
# full-sib used as calibration set. This is the difference between the full-sib to
# serve as calibration set and all full-sibs in a specific bi-parental population

ValSet = vector("list", length(Inds_in_a_POP) - 1)
```

```r
# Define the Validation Set for each calibration set
# which is the difference between the full-sib
# to serve as calibration set and all full-sibs in a specific
# bi-parental population.

for (j in 1:length(Inds_in_a_POP)) {
ValSet[[j]] = setdiff(Inds_in_a_POP, Inds_in_a_POP[j])

}


# Calculate the CDmean for each bi-parental population

for (m in 1:length(Inds_in_a_POP)) {
# Combine each validation set and the corresponding calibration set

tst_trn_Raw = c(ValSet[[m]], Inds_in_a_POP[m])


# Set the size of the validation and calibration set
tst_size = length(ValSet[[m]])

trn_size = length(Inds_in_a_POP[m])

# Get the vector of the calibration and validation set
trn = Inds_in_a_POP[m]
tst = ValSet[[m]]


# Combine the calibration and validation set
tst_trn = c(tst, trn)


# Get the genomic relatonship matrix of the tst_trn
Gmatrix_new = Gmatrix[tst_trn, tst_trn]

# Get the inverse of the Gmatrix_new
if (isTRUE(is.non.singular.matrix(Gmatrix_new))) {
invAt_new = solve(Gmatrix_new)

      } else {

      invAt_new = ginv(Gmatrix_new)

      }


# TT is the matrix of contrasts, each column is a contrast
# between of the validation set and the mean of the
# entire population (tst_trn). That is of dimension:
# total number of individuals in a bi-parental population
# number of individuals as validation set.
```

```r
TT = matrix(0, length(rownames(Gmatrix_new)), tst_size)
TT[1:tst_size, ] = -1 / tst_size
for (i in 1:tst_size) {
TT[i, i] = 1 - 1 / tst_size

}

dim(TT)

# Get the design matrix for the calibration set
X_trn <- rep(1, trn_size)
Ident_trn <- diag(trn_size)
M_trn <-
Ident_trn - (X_trn %*% solve(t(X_trn) %*% X_trn) %*% t(X_trn))
dim(M_trn)

# Design matrix indicating the individual used as the calibration set
Z_trn = matrix(0, trn_size, trn_size + tst_size)

for (i in 1:trn_size) {
Z_trn[i, tst_size + i] = 1
}

dim(Z_trn)

lambda = 1

# compute the CDs of the contrasts
if (isTRUE(is.non.singular.matrix(Gmatrix_new))) {
   RawCD <-
   (t(TT) %*% (
   Gmatrix_new - lambda * solve(t(Z_trn) %*% M_trn %*% Z_trn + lambda * invAt_new)
   ) %*% TT) / (t(TT) %*% Gmatrix_new %*% TT)

   } else {

   RawCD <-
   (t(TT) %*% (
   Gmatrix_new - lambda * ginv(t(Z_trn) %*% M_trn %*% Z_trn + lambda * invAt_new)
   ) %*% TT) / (t(TT) %*% Gmatrix_new %*% TT)
   CD = diag(RawCD)

   }

CD = diag(RawCD)

# CD value of the initial training set
CDmeanSave = mean(CD)

CDmean$CDmean[m] = CDmeanSave

}
```

```
    CDmean <- CDmean[order(-CDmean$CDmean),]

    TRNoptimized_E1 <-
    CDmean[order(-CDmean$CDmean)[1:NTrnSample], "GID"]

    Output_E1[[s]] = TRNoptimized_E1

    TRNoptimized_E2 = CDmean[order(-CDmean$CDmean)[(NTrnSample + 1):length(tst_trn)], "GID"]

    Output_E2[[s]] = TRNoptimized_E2

    Output_E3 [[s]] = c(TRNoptimized_E1[1:ceiling(NTrnSample / 2)],
                        TRNoptimized_E2[1:ceiling(NTrnSample /2)])


    }



    return(save(Output_E1, Output_E2, Output_E3, file = "TRN_E1_E2_E3.RData"))


}
```
